# A System Dynamics Model to Predict the Human Monocyte Response to Endotoxins

**DOI:** 10.3389/fimmu.2017.00915

**Published:** 2017-08-03

**Authors:** Enrique Álvarez, Víctor Toledano, Fernando Morilla, Enrique Hernández-Jiménez, Carolina Cubillos-Zapata, Aníbal Varela-Serrano, José Casas-Martín, José Avendaño-Ortiz, Luis A. Aguirre, Francisco Arnalich, Charbel Maroun-Eid, Alejandro Martín-Quirós, Manuel Quintana Díaz, Eduardo López-Collazo

**Affiliations:** ^1^Innate Immunity Group, IdiPAZ, La Paz University Hospital, Madrid, Spain; ^2^EMPIREO S.L., Madrid, Spain; ^3^Tumor Immunology Laboratory, IdiPAZ, La Paz University Hospital, Madrid, Spain; ^4^Center for Biomedical Research Network, CIBERES, Madrid, Spain; ^5^Department of Information Technology and Automation, ETSI Information Technology, National University of Distance Learning UNED, Madrid, Spain; ^6^Internal Medicine Service, La Paz University Hospital, Madrid, Spain; ^7^Department of Emergency, La Paz University Hospital, Madrid, Spain

**Keywords:** monocytes, endotoxin tolerance, inflammation, bacterial lipopolysaccharide, mathematical model

## Abstract

System dynamics is a powerful tool that allows modeling of complex and highly networked systems such as those found in the human immune system. We have developed a model that reproduces how the exposure of human monocytes to lipopolysaccharides (LPSs) induces an inflammatory state characterized by high production of tumor necrosis factor alpha (TNFα), which is rapidly modulated to enter into a tolerant state, known as endotoxin tolerance (ET). The model contains two subsystems with a total of six states, seven flows, two auxiliary variables, and 14 parameters that interact through six differential and nine algebraic equations. The parameters were estimated and optimized to obtain a model that fits the experimental data obtained from human monocytes treated with various LPS doses. In contrast to publications on other animal models, stimulation of human monocytes with super-low-dose LPSs did not alter the response to a second LPSs challenge, neither inducing ET, nor enhancing the inflammatory response. Moreover, the model confirms the low production of TNFα and increased levels of C–C motif ligand 2 when monocytes exhibit a tolerant state similar to that of patients with sepsis. At present, the model can help us better understand the ET response and might offer new insights on sepsis diagnostics and prognosis by examining the monocyte response to endotoxins in patients with sepsis.

## Introduction

Endotoxin tolerance (ET) has been described as a transient state in which monocytes/macrophages are refractory to further stimulation with endotoxins such as lipopolysaccharides (LPSs), the major component of the outer membrane of Gram-negative bacteria (1, 2). ET has been studied in detail both *in vitro* and *in vivo* in animal models as well as in humans (3–6). This phenomenon takes place in several clinical situations, such as sepsis, in which the monocytes isolated from patients show a reduced production of proinflammatory cytokines in response to an *ex vivo* endotoxin challenge (7, 8). ET has also been reported in patients with acute coronary syndrome (9), cystic fibrosis (6, 10, 11), chronic lymphocytic leukemia (12), and stroke (13).

Once the endotoxin is recognized by the receptors on the host monocyte/macrophage lineage, a signaling cascade is triggered, resulting in the rapid expression of specific proinflammatory cytokines such as tumor necrosis factor alpha (TNFα) interleukin-12, (IL-12), IL-6, and IL-1β, and chemokines such as C–C motif ligand 2 (CCL2) and CXC motif ligand 12. However, the inflammatory response must be regulated to prevent damaging systemic inflammation, also known as a “cytokine storm.” Thus, after the first wave of proinflammatory cytokines, the monocytes are functionally reprogrammed to produce cytokines with anti-inflammatory properties, such as IL-10 and transforming growth factor β (TGF-β) (1, 2). The plasticity of these cells allows changes in the gene expression signatures that can be considered as various proinflammatory and anti-inflammatory phenotypes (14, 15). The anti-inflammatory phenotype and ET have been shown to be highly related and are orchestrated by common signaling pathways (16). Although CCL2 is primarily implicated in the recruitment of monocytes/macrophages to the inflammatory site (17, 18), it is highly expressed in the monocytes from patients with sepsis, who show marked ET (19). In contrast, a distinct effect known as potentiation, in which the cells show an increased expression of proinflammatory cytokines in response to a second LPSs challenge, has been reported, particularly in mouse models (20–22). In humans, however, this phenomenon appears to be absent or weaker, probably because there are several differences in the inflammatory response to endotoxins between mice and humans (e.g., a higher endotoxin challenge is necessary in mice to achieve a similar response as is achieved in humans) (23, 24).

System dynamics has been proven to be a powerful instrument for analyzing social, economic, ecological, and biological systems (25, 26), offering computerized models that allow systematic testing of various scenarios. Mathematical models have been previously used to study the inflammatory response, ET, and sepsis (27–30). However, these models were developed and tested using datasets from experiments using animals such as mice, rats, and swine (28, 29, 31, 32). Thus, the models might not be valid for humans, especially in regard to the differences in sensitivity to endotoxins and the potentiation phenomenon. To investigate the response of humans to endotoxins in depth, we modeled monocyte responses to endotoxins and their progression to an ET state using system dynamics. The model was tested using datasets obtained from experiments using human cells. The developed model is able to reproduce several real-life situations, such as the ET status of monocytes in patients with sepsis.

## Materials and Methods

### Modeling

**Figure 1:**
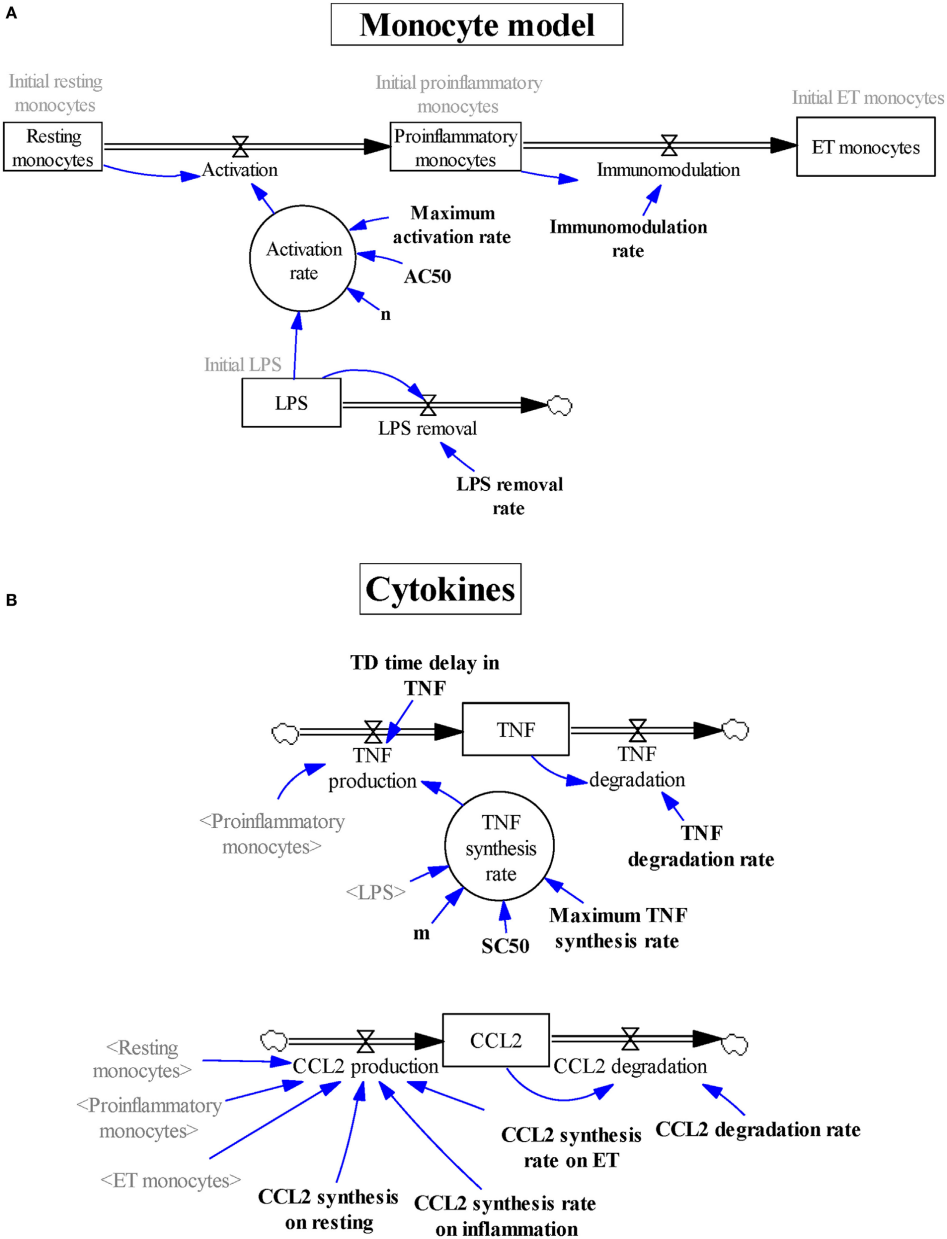
Graphical description using Vensim software of the subsystems describing **(A)** the various subsets of monocytes and **(B)** the levels of tumor necrosis factor and CCL2. Stock variables are represented inside boxes; flow variables are shown as double lines with an arrow and a valve symbol; the rest of the variables are auxiliary variables (inside circles), shadow variables (in gray between angled brackets), parameters (in bold), or initialization values (in gray); single lines with an arrow are used to specify that the destination variable is affected by the origin variable.

**Figure 2:**
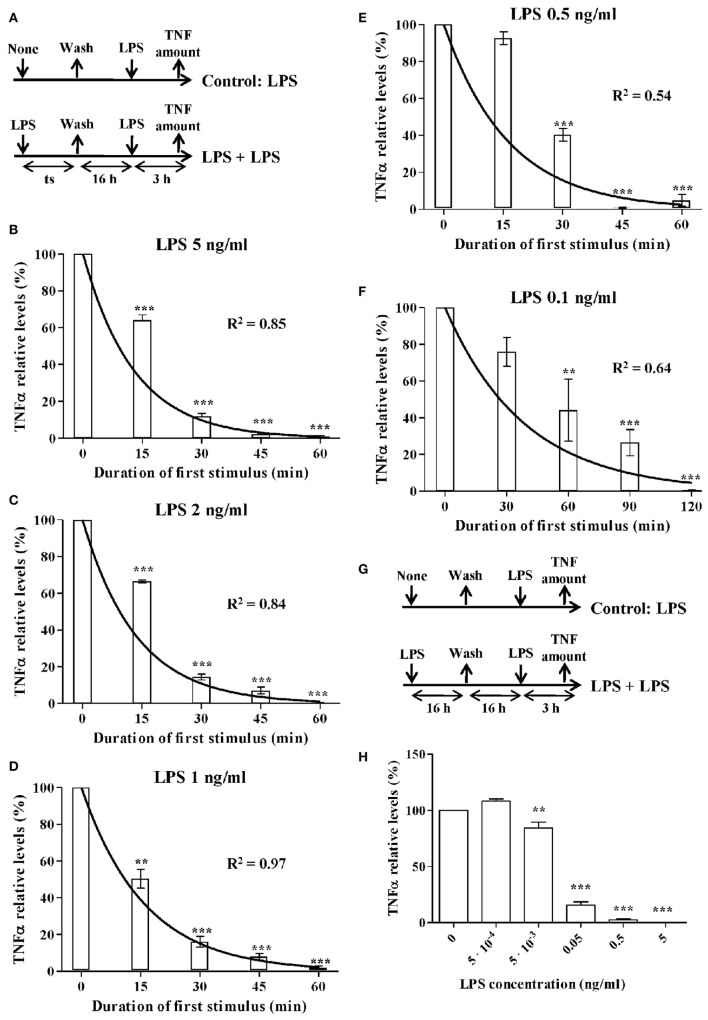
The activation rate of monocytes treated with lipopolysaccharides (LPSs). **(A)** Schematic representation of the endotoxin tolerance (ET) model used. Human monocytes isolated from PMBCs were pretreated with **(B)** 5 ng/ml, **(C)** 2 ng/ml, **(D)** 1 ng/ml, **(E)** 0.5 ng/ml, or **(F)** 0.1 ng/ml LPS for the indicated times (time of stimulation, *t_s_*), washed twice with PBS, cultured in medium for 16 h, and restimulated with 5 ng/ml LPS for 3 h. The supernatants were then harvested and the tumor necrosis factor alpha (TNFα) levels were evaluated by cytometric bead array (CBA). Relative TNFα levels to control monocytes without LPS pretreatment (treated only with the second LPS stimulus at 5 ng/ml), were expressed as mean ± SEM (*n* = 3 healthy volunteers). **p < 0.01; ***p < 0.001 compared with the value for the control as assessed by ANOVA and Dunnett’s Multiple Comparison post-test. Curves fitting the experimental data were obtained by non-linear regression. The coefficient of determination *R*^2^ is presented in each case to measure the goodness of the adjustment. **(G)** Schematic representation of the ET model used for low-LPS doses. **(H)** Human monocytes isolated from PMBCs were pretreated with the indicated LPS concentrations for 16 h, washed twice with PBS, cultured in medium for 16 h, and restimulated with 5 ng/ml LPS for 3 h. The supernatants were then harvested and the TNFα levels were evaluated by CBA. Relative TNFα levels to control monocytes without LPS pretreatment (treated only with the second LPS stimulus at 5 ng/ml), were expressed as mean ± SEM (*n* = 3 healthy volunteers). **p < 0.01; ***p < 0.001 compared with the value for the control as assessed by ANOVA and Dunnett’s Multiple Comparison post-test.

**Figure 3:**
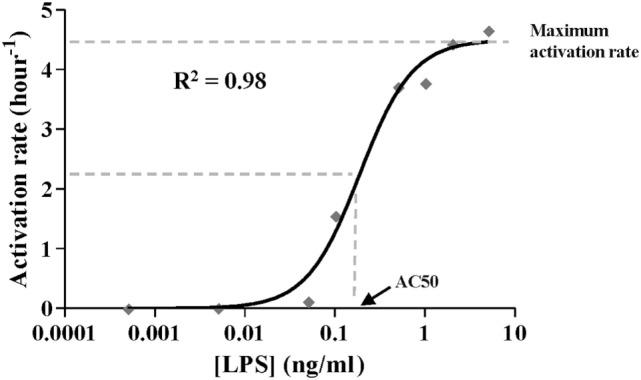
Activation rate expressed as Hill function. The black line corresponds to the function optimized by dose–response non-linear regression analysis to fit estimated values (gray diamonds) shown in Table 5. The coefficient of determination *R*^2^ is presented. [LPS] refers to the lipopolysaccharides concentration in the first stimulus.

**Figure 4:**
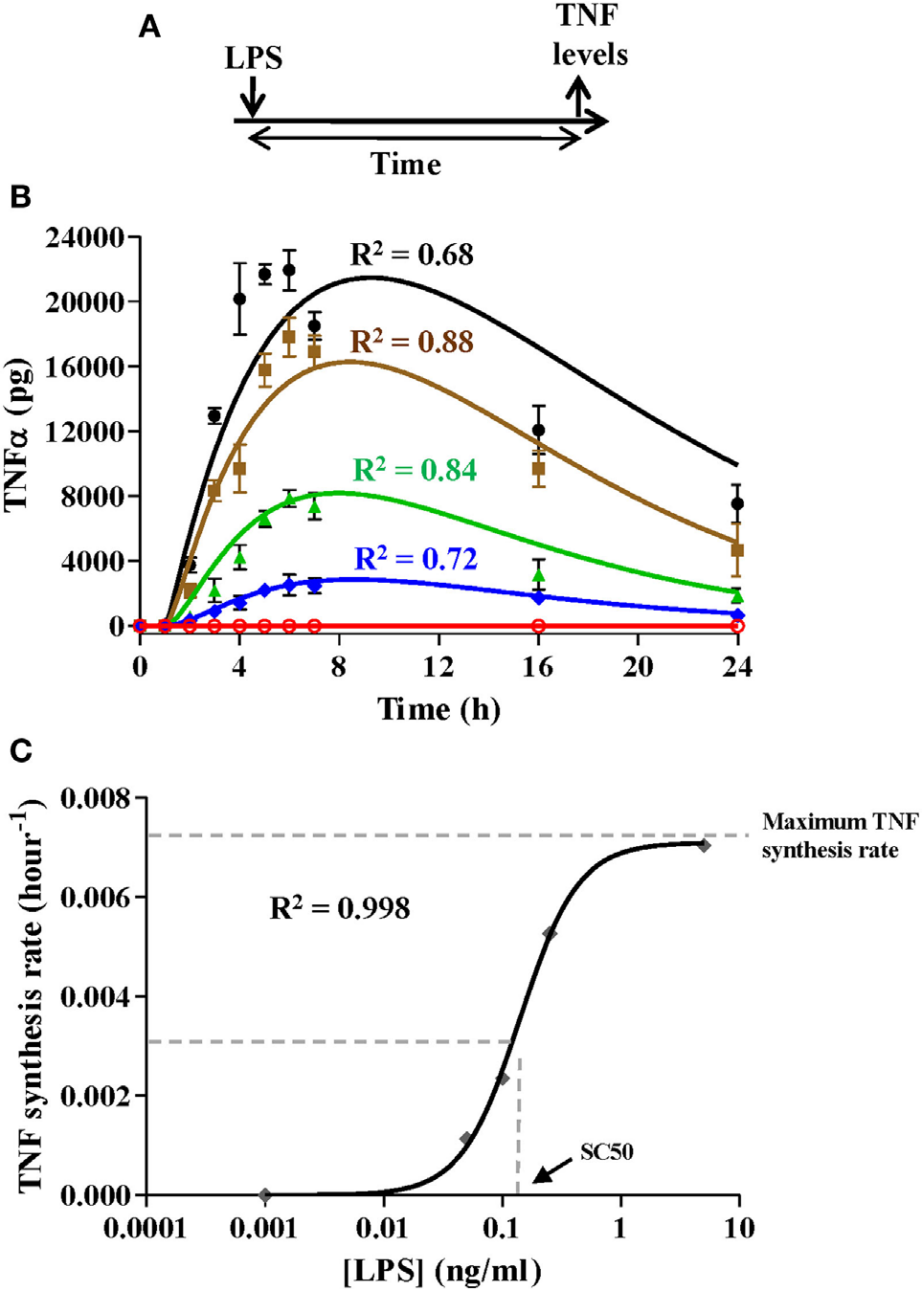
Simulation of various proinflammatory scenarios. Experimental data were obtained from cultures of 10^6^ monocytes isolated from healthy volunteers challenged with lipopolysaccharides (LPSs) as described in **(A)** at a concentration of 5, 0.25, 0.1, 0.05 ng/ml, or not challenged. Tumor necrosis factor (TNFα) levels were quantified in the monocyte cultures at the indicated times using cytometric bead array assays and were graphically represented **(B)** as black circles (LPSs 5 ng/ml), brown squares (LPSs 0.25 ng/ml), green triangles (LPSs 0.1 ng/ml), blue diamonds (LPSs 0.05 ng/ml), or red circles (not challenged). Real data were expressed as the mean ± SEM (*n* = 3 healthy volunteers). The model was programmed with 10^6^ monocytes in the resting monocyte state and the same initial LPSs concentrations. Outputs from the model simulation for TNF state were graphically represented **(B)** as black line, LPSs 5 ng/ml; brown line, LPSs 0.25 ng/ml; green line, LPSs 0.1 ng/ml; blue line, LPSs 0.05 ng/ml; or red line, not challenged. The coefficient of determination *R*^2^ is presented in each case to measure the goodness of the adjustment. **(C)** Adjustment of the TNF synthesis rate Hill function. The black line corresponds to the function optimized to fit the estimated TNF synthesis rates from Table 6. [LPS] refers to the LPSs concentration in the medium.

**Figure 5:**
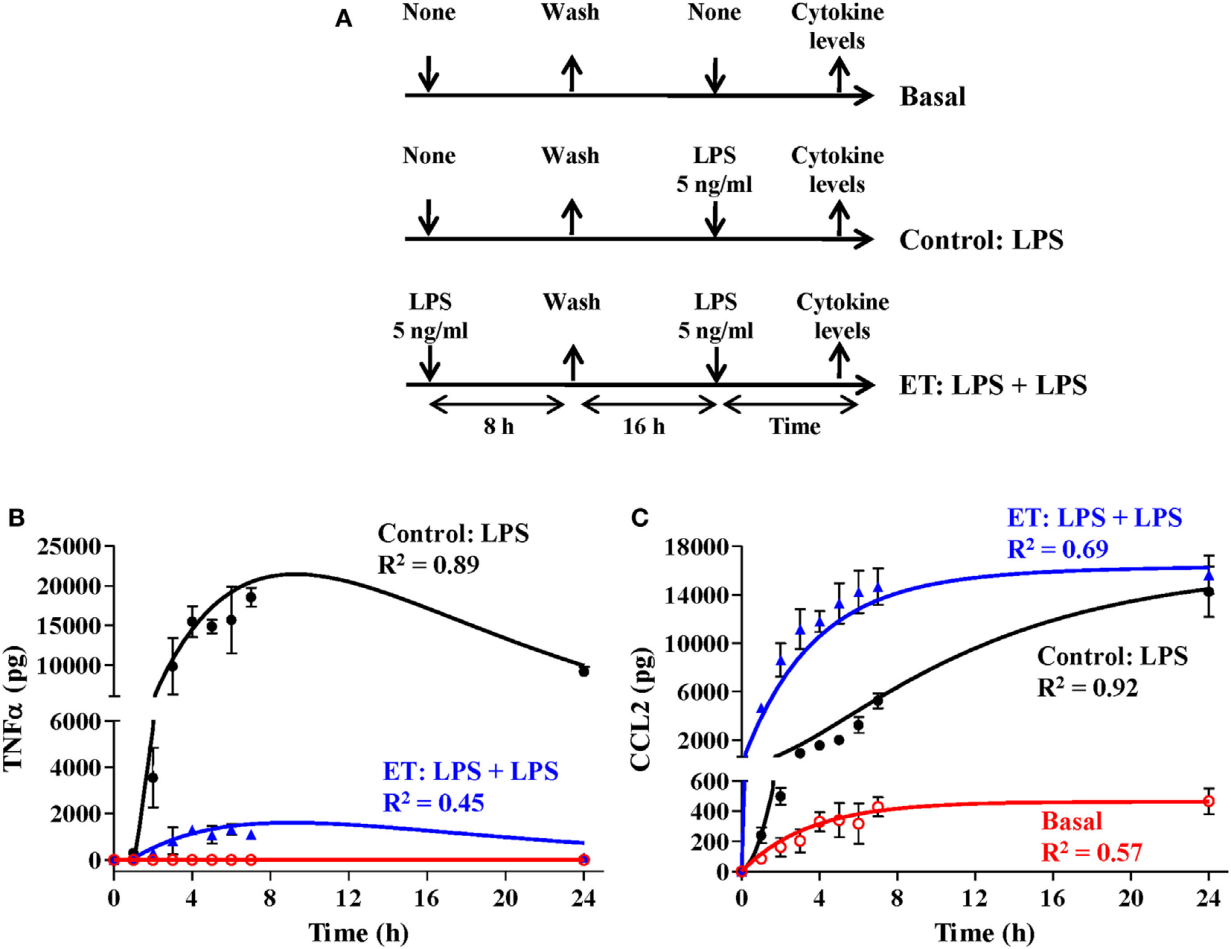
Simulation of an endotoxin tolerance (ET) scenario. **(A)** Schematic representation of the ET model used for this study. The cultures of human monocytes were or not pretreated with 5 ng/ml LPSs for 8 h, washed twice with PBS, cultured in medium for 16 h, and restimulated with 5 ng/ml lipopolysaccharides. Supernatants were harvested at the indicated times, tumor necrosis factor alpha (TNFα) **(B)**, and C–C motif ligand 2 (CCL2) **(C)** protein levels were evaluated by cytometric bead array and then graphically represented as black circles (control), blue triangles (ET), or red circles (basal). Real data were expressed as means experiments ± SEM (*n* = 3 healthy volunteers). To simulate the ET scenario, the model was programmed with 0, 75,000, and 925,000 monocytes in the resting monocytes state, the proinflammatory monocytes state, and the ET monocytes state, respectively. The control scenario was simulated as described in Figure 4. Outputs from TNF **(B)** and CCL2 **(C)** states were graphically represented as black line (control), blue line (ET), or red line (basal). The coefficient of determination *R*^2^ was calculated in each case to measure the goodness of the adjustment and is presented in the graphs.

**Figure 6:**
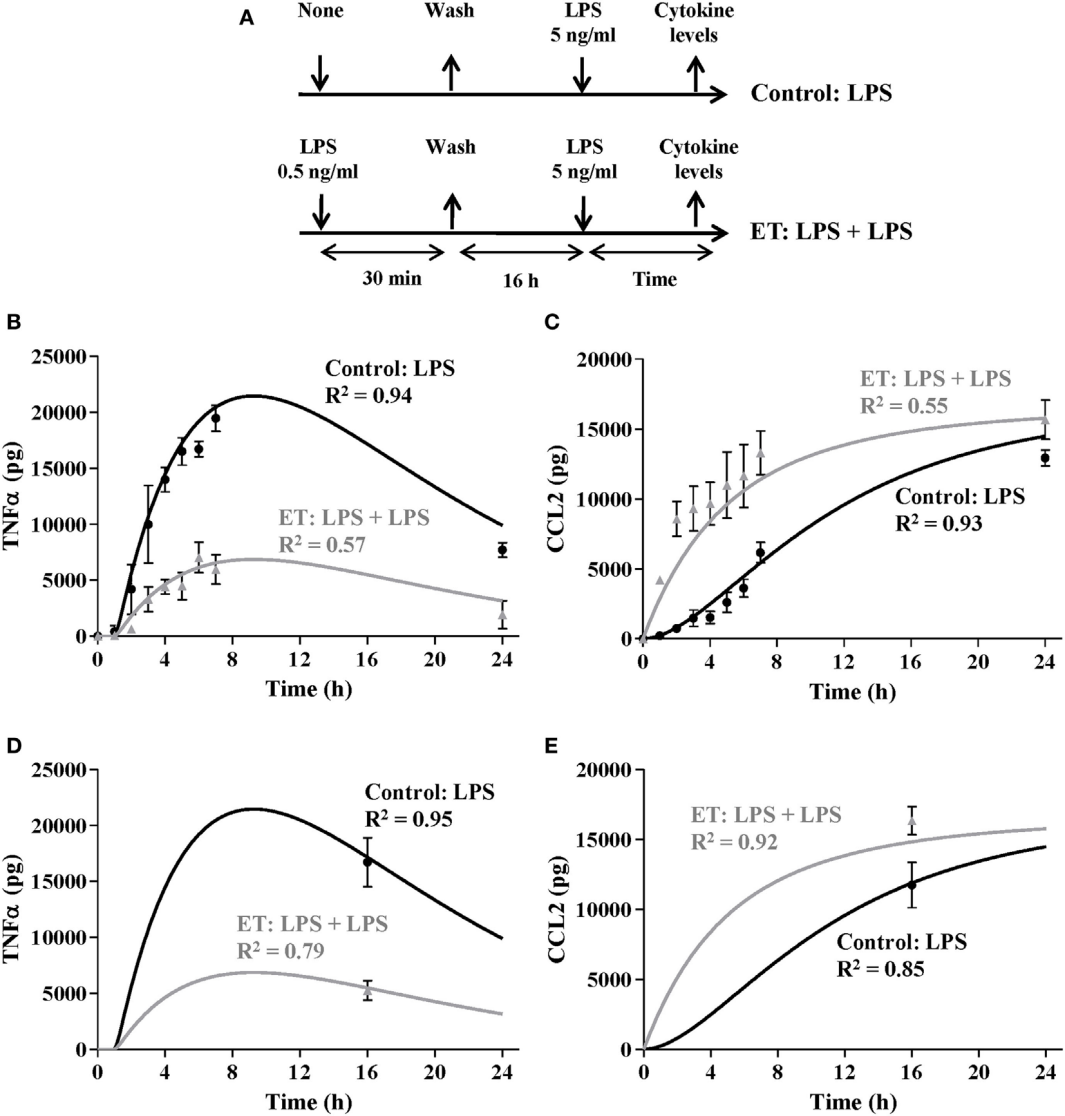
Validation of the model by simulating endotoxin tolerance (ET) scenarios. **(A)** Schematic representation of the ET model used for this study. The cultures of human monocytes were or not pretreated with 0.5 ng/ml lipopolysaccharides (LPSs) for 30 min, washed twice with PBS, cultured in medium for 16 h, and restimulated with 5 ng/ml LPSs. Supernatants were harvested at the indicated times, tumor necrosis factor alpha (TNFα) **(B)**, and C–C motif ligand 2 (CCL2) **(C)** protein levels were evaluated by cytometric bead array and then graphically represented as black circles (control) or gray triangles (ET). Real data were expressed as mean ± SEM (*n* = 3 healthy volunteers). The model was programmed with a total 10^6^ monocytes and challenged with 5 ng/ml LPS. The Tolerance index was adjusted by an optimization process to 68% to minimize the sum of the standard quadratic errors for TNF and CCL2 simulations. Outputs for TNF **(B)** and CCL2 **(C)** states were graphically represented (gray lines). For comparison, simulation of 10^6^ monocytes with a tolerance index equals to 0 and challenged with the same LPSs concentration was also represented as black lines. Cultures of 10^6^ monocytes from patients with sepsis were stimulated with 5 ng/ml LPS, and TNF **(D)** and CCL2 **(E)** levels were measured after 16 h and then graphically represented as gray triangles. As control the results with 10^6^ monocytes from healthy volunteers are also represented (black circles). Real data were expressed as mean ± SEM (*n* = 3). The model was programmed with a total 10^6^ monocytes and challenged with 5 ng/ml LPS. The Tolerance index was adjusted as described above to 68%. Outputs for TNF **(D)** and CCL2 **(E)** states were graphically represented as gray lines. For comparison, simulation of 10^6^ monocytes with a tolerance index equals to 0 and challenged with the same LPS concentration was also represented as black lines.

The model was developed following the four-step sequence proposed by the system dynamics methodology (25). First, experimental data and other evidence were used to create a mental modeling of the response of monocytes to endotoxins. Second, the model structure able to explain the polarization of the monocytes from the proinflammatory to the ET phenotype was represented as a Forrester diagram (Figure 1A). Third, the system was mathematically modeled as a continuous dynamic process represented by a set of differential and algebraic equations (Tables 1–3). Finally, the model was parameterized (Table 4), optimized, and validated to fit the experimental data from Figures 2–6 and findings published in previous articles (6, 33, 34). The parameters of the Hill function corresponding to the activation rate were adjusted by using a non-linear regression analysis (Figure 3) to fit the experimental data summarized in Table 5. Similarly, the parameters of the Hill function corresponding to the TNF synthesis rate were adjusted by using a non-linear regression analysis (Figure 4C) to fit the estimated TNF synthesis rates for each LPSs concentration and summarized in Table 6. The rest of the model parameters were adjusted using the Vensim’s optimizer and Matlab to get the best match between model behavior and the data (Figures 4B and 5C). These adjustments were performed by minimizing the total quadratic error, which measures the goodness of the model. Finally, the model was validated using datasets from Figures 5B and 6.

**Table 1:** Differential equations of the model stocks.

Equation no.	Stock	Equation	Units
1	Resting monocytes	$\frac{d\text{Resting monocytes}(t)}{dt}=-\text{Activation}(t)$	Monocytes
2	Proinflammatory monocytes	$\frac{d\text{Proinflammatory monocytes}(t)}{dt}=\text{Activation}(t)–\text{Immunomodulation}(t)$	Monocytes
3	ET monocytes	$\frac{d\text{ET monocytes}(t)}{dt}=\text{Immunomodulation}(t)$	Monocytes
4	LPS	$\frac{d\text{LPS}(t)}{dt}=-\text{LPS removal}(t)$	Nanogram
5	TNF	$\frac{d\text{TNF}(t)}{dt}=\text{TNF production}(t)-\text{TNF degradation}(t)$	Picogram
6	CCL2	$\frac{d\text{CCL2}(t)}{dt}=\text{CCL2 production}(t)–\text{CCL2 degradation}(t)$	Picogram

**Table 2:** Algebraic equations of the model flows.

Equation no.	Flow	Equation	Units
1	Activation	Activation(t)= Resting monocytes(t)· Activation rate(t)	Monocytes per hour
2	Immunomodulation	Immunomodulation(t)=Proinflammatory monocytes(t)⋅Immunomodulation rate	Monocytes per hour
3	LPS removal	LPS removal(t)= LPS(t)· LPS removal rate	Nanogram per milliliter per hour
4	TNF production	TNF production(t) = Proinflammatory monocytes(t−TD time delay in TNF)⋅ TNF synthesis rate(t)	Picogram per hour
5	TNF degradation	TNF degradation(t)=TNF(t)· TNF degradation rate	Picogram per hour
6	CCL2 production	$\begin{array}{l} \text{CCL2 production}(t)=\text{Resting monocytes}(t)\cdot \text{CCL2 synthesis rate on resting + Proinflammatory}\\ \text{monocytes}(t)\cdot \text{CCL2 synthesis rate on inflammation + ET monocytes}(t)\cdot \text{CCL2 synthesis rate on ET} \end{array}$	Picogram per hour
7	CCL2 degradation	CCL2 degradation(t)=CCL2(t)· CCL2 degradation rate	Picogram per hour

**Table 3:** Algebraic equation for the model auxiliary variable.

Equation no.	Auxiliary variable	Equation	Units
1	Activation rate	$\text{Activation rate}(t)=\frac{\text{Maximum activation rate}\cdot \text{ LPS}{\left(t\right)}^{n}}{\text{AC}50^{n}+\text{LPS}{\left(t\right)}^{n}}$	Per hour
2	TNF synthesis rate	$\text{TNF synthesis rate}(t)=\frac{\text{Maximum TNF synthesis rate}\cdot \text{ LPS}{\left(t\right)}^{m}}{\text{SC}50^{m}+\text{LPS}{\left(t\right)}^{m}}$	Per hour

**Table 4:** Model parameters and their values.

*N*	Parameter	Value	Units
1	Maximum activation rate	4.4990	Per hour
2	AC50	0.1889	Nanogram per milliliter
3	n	1.4825	Dimensionless
4	Immunomodulation rate	0.1088	Per hour
5	LPS removal rate	0.0726	Per hour
6	Maximum TNF synthesis rate	0.0071	Picogram per hour per monocyte
7	SC50	0.0890	Nanogram per milliliter
8	m	1.7670	Dimensionless
9	TD time delay in TNF	1	Hour
10	TNF degradation rate	0.1362	Per hour
11	CCL2 synthesis rate on resting	0.1315 · 10^−3^	Picogram per hour per monocyte
12	CCL2 synthesis rate on inflammation	0.1315 · 10^−3^	Picogram per hour per monocyte
13	CCL2 synthesis rate on ET	0.4633 · 10^−2^	Picogram per hour per monocyte
14	CCL2 degradation rate	0.2828	Per hour

**Table 5:** Empirical activation rates depending on the amount of lipopolysaccharide (LPS).

LPS (ng/ml)	Activation rate (h^−1^)
5	4.6581
2	4.4318
1	3.7784
0.5	3.7100
0.1	1.5527
0.05	0.1177
0.005	0.0124
0.0005	0.0000

**Table 6:** Estimated tumor necrosis factor synthesis rates depending on the amount of lipopolysaccharides (LPSs).

LPS (ng/ml)	Activation rate (h^−1^)
5	0.7043 · 10^−2^
0.25	0.5270 · 10^−2^
0.1	0.2359 · 10^−2^
0.05	0.1136 · 10^−2^
0.001	0.0000

The modeling process, simulations, and optimization analyses were performed using Vensim DSS software, version 5.7a (Ventana Systems, Harvard, MA, USA) and Matlab R2015a (Mathworks, Inc., MA, USA).

### Patients

Peripheral blood was obtained from three patients with sepsis (mean age ± SD: 68 ± 10.6 years) who had microbiologically confirmed Gram-negative bacteremia (positive blood cultures for *Escherichia coli*), secondary to a urinary tract infection. The patients who met the consensus conference definition of sepsis (35) were admitted to the Department of Internal Medicine Service at La Paz University Hospital. Blood samples were collected from the patients within 24 h of sepsis confirmation by blood culture. The following exclusion criteria were imposed: the presence of malignancy or chronic inflammatory diseases, treatment with steroids or immunosuppressive drugs during the last month, hepatic failure (serum aspartate aminotransferase and/or alanine aminotransferase > 100 IU/l; prothrombin time < 60%; total bilirubin < 60 μmol/l), renal insufficiency (plasma creatinine > 200 μmol/l), HIV/AIDS, hepatitis B or C, pregnancy, and age > 80 years. Peripheral blood from 18 healthy volunteers was also obtained. Of them, 12 were used in setting up the model in Figures 2, 4 and 5, and the other 6 were used in validating the model in Figure 6. All the procedures were in accordance with the Helsinki Declaration of 2000, and informed consent was obtained from all the participants. This study was approved by the La Paz University Hospital Ethics Committee.

### Peripheral Blood Mononuclear Cells (PBMCs), Monocyte Isolation, Cell Culture, and Reagents

Peripheral blood mononuclear cells were isolated from healthy volunteers or sepsis patients by centrifugation on Ficoll-Hypaque Plus (Amersham Biosciences), and monocytes were obtained by adherence as previously described (6). The purity of the monocyte cultures was tested by CD14 labeling and flow cytometry analysis (average 82% CD14 + cells). Other cell surface markers were also tested (average CD1a = 4.1%, and CD89 = 85%). All the reagents used for the cell cultures were endotoxin-free, as assayed with the limulus amebocyte lysate test (Cambrex).

To establish the model parameters, the monocytes were treated with LPS concentrations ranging from 5 ng/ml to 0.5 pg/ml during the times of stimulation (*t_s_*) indicated in Figure 2. After LPS treatment, the cells were washed three times with PBS and kept in complete medium for 16 h to ensure the monocytes entered into ET. The cells were then restimulated with 5 ng/ml LPS for 3 h and cytokine levels were quantified. The control cells were not treated with LPS during the first stimulation.

To compare the model output with experimental data, monocytes from healthy volunteers and patients with sepsis were treated once or twice with LPSs at the concentrations indicated in Figures 4–6. Samples of culture supernatant were collected at times ranging from 1 to 24 h to quantify the levels of TNFα and CCL2.

The LPS from *Salmonella abortus* was a kind gift from Dr. Galanos (Max Planck Institute for Immunobiology, Freiburg, Germany). All work with LPS was performed in biosafety level (BSL) two facilities under appropriate work practices, and proper use of personal protective equipment. The medium used for the cell culture was DMEM from Invitrogen.

### Cytometric Bead Array (CBA)

The cytokine levels (TNFα and CCL2) in the culture supernatants from the human samples were determined using the CBA Flex Set (BD Biosciences) following the manufacturer’s protocol. The data collected were analyzed by flow cytometry, using a BD FACSCalibur flow cytometer (BD Biosciences).

### Statistical Analysis of Experimental Data

The data were collected and are expressed as mean ± SEM (*n* is indicated in each figure legend). The statistical significance was set at *p* < 0.05 and all statistical analyses were conducted using Prism 5.0 software (GraphPad) and Matlab.

## Results

### Modeling

A dynamic compartmental model can be used to properly parameterize and describe several components of the immune system (36, 37). In this sense, both physical sites and functions, such as the organs of the immune system and states of immune cell differentiation, respectively, can be schematized as a set of compartments.

To explain the transition of the peripheral blood monocytes through the various functional stages after an endotoxin challenge, we considered a dynamic model divided into two subsystems (Figures 1A,B). The main subsystem reproduces the activation/immunomodulation process by which monocytes transit after endotoxin challenge. It contains four compartments (states) and three transitions (physical flows) between them (Figure 1A), which are based on various assumptions. First, the monocytes are classified into three subgroups: resting monocytes, proinflammatory monocytes, and ET monocytes. Second, the transition from the resting monocytes to the proinflammatory monocytes (activation) only occurs when LPSs are present in the medium. Third, the flow between proinflammatory monocytes and ET monocytes (immunomodulation) is an LPS-independent process. Finally, we assumed that LPS concentration is not continually the same; thus, an outflow was included in the model (LPS removal).

The model also contains a second subsystem modeling the production of TNF (as a proinflammatory marker) and CCL2 (as an ET marker) (Figure 1B). Because these cytokines are differentially synthesized by the three subsets of monocytes, this subsystem is used to indirectly monitor the dynamic process followed by monocytes modeled in the main subsystem. It is composed of two states (TNF and CCL2) and four flows (TNF production, TNF degradation, CCL2 production, and CCL2 degradation), modifying the balance of these stocks.

The model structure, built with Vensim software (Figures 1A,B), contains the states and flows previously mentioned, two auxiliary variables (inside circle), and 14 parameters (in bold). These elements are linked by the physical flows (double line with arrow) and by the information transmissions (single line with arrow), according to the mathematical model represented by the set of differential and algebraic equations (Tables 1–3).

The six differential equations of Table 1 establish the mass balance (inflows minus outflows) in the compartments. The first three differential equations of Table 1 describe the changes in the number of monocytes in the three subgroups. Because LPS concentration in the medium is known to decrease over time, changes in LPSs are also represented by a differential equation (Table 1, Eq. 4). Equations 5 and 6 of Table 1 express the changes in TNF and CCL2 levels per time unit, respectively.

The activation of monocytes after LPS stimulation depends directly on the stock of resting monocytes and the auxiliary variable activation rate (Table 2, Eq. 1). The activation rate is modulated by a Hill activation function (Table 3, Eq. 1). This expression depends on: the maximum activation rate, which allows setting the top value that the auxiliary variable can take; the AC50, which sets the concentration of LPS needed to reach 50% of the maximum activation rate; the *n* Hill coefficient, which describes the steepness of the curve; and the LPS concentration expressed as a stock variable. The immunomodulation depends directly on the amount of proinflammatory monocytes and the parameter immunomodulation rate (Table 2, Eq. 2). Similarly, LPS removal at a given time is directly proportional to the LPS concentration at that time and the LPS removal rate parameter (Table 2, Eq. 3).

Equation 4 of Table 2 expresses the TNF production that depends on the amount of proinflammatory monocytes, the auxiliary variable TNF synthesis rate, and the TD time delay in TNF parameter. Because the signaling cascade initiated after the binding of LPSs to the receptors in the plasmatic membrane leads to a delay between the time in which the resting monocytes are being activated and the production of TNF, the parameter TD time delay in TNF was incorporated to model this fact. In addition, TNFα expression in response to LPSs is regulated by the translocation of NF-κB to the nucleus. NF-κB translocation is highly dependent on the LPS concentration in the medium and it has been shown that the LPS-dose–response curve of NF-κB translocation follows a Hill equation (34). Therefore, in the model the TNF synthesis rate is modulated by a Hill activation function (Table 3, Eq. 2). This expression depends on: the maximum TNF synthesis rate, which is the top value that the auxiliary variable can take; the SC50 which sets the concentration of LPS needed to reach half of the maximum TNF synthesis rate; the *m* Hill coefficient which defines the slope of the curve; and the LPS concentration.

Because CCL2 is produced by the three subtypes of monocytes at different rates, CCL2 production is represented by an equation that considers the three states, each of which is multiplied by its corresponding parameter (Table 2, Eq. 6). The TNF degradation and CCL2 degradation outflows are proportional to the mass in the source stock (TNF and CCL2, respectively) and the TNF degradation rate or CCL2 degradation rate parameters, respectively (Table 2, Eqs 5 and 7, respectively).

### Parameterization, Optimization, and Validation of the Model

A model’s performance is highly dependent on its parameters. Thus, defining the values of the model’s parameters is one of the most important processes in system dynamics modeling. Although this model is relatively simple, the parameterization was challenging due to the uncertainty of the parameter values. As a consequence, there is a wide range of value combinations, many of which can result in unexpected model outputs far from the reality. To solve this problem, the parameters were sequentially estimated by isolating the different processes (e.g., activation, TNF production, CCL2 production, etc.) from the rest. Thus, the values were estimated by using the experimental data from Figures 2–5. They were optimized following various approaches, such as non-linear regression analysis, the Vensim’s optimization tool, or using discrete models in Matlab to set the final values for the model parameters that are summarized in Table 4. These approaches require to select initial values and ranging values for some parameters that are not being optimized in that moment. To this end, findings published in previous articles were used (6, 33, 34).

As described above, the Hill function to express the activation rate depends on the maximum activation rate, the AC50, and *n* parameters. To obtain the value for these three parameters, the activation rate was estimated for monocytes from healthy volunteers using various LPSs concentrations and following the experimental design described in Figure 2A. Since TNF is produced by the proinflammatory subset of monocytes, this cytokine was used to indirectly estimate the percentage of cells that transit to the proinflammatory state after an LPSs challenge. Thus, the relative TNFα expression levels after a second LPSs challenge with 5 ng/ml LPS were used to estimate the percentage of resting monocytes that were not activated during the first stimulus (Figures 2B–F). As expected, when the time of the first challenge with LPS increased, there was a decrease in the fraction of cells remaining in the resting state after the first challenge (Figures 2B–F). In addition, this decrease was faster when the LPS concentrations were higher (compare Figures 2B,F). The activation rates for each LPSs concentration were estimated from the adjusted one-step decay functions obtained by non-linear regression analysis of the experimental data (Figures 2B–F) and are summarized in Table 5. Since the activation rates at super-low LPS doses are longer, the experimental design was slightly changed. Thus, the monocytes were first challenged with increasing concentrations of LPS, ranging from 0.5 pg/ml to 5 ng/ml, following the scheme of Figure 2G. The first stimulus was maintained for 16 h to significantly reduce the relative expression of TNFα in the second challenge with 5 ng/ml LPS. The percentage of resting monocytes that were not activated during the first stimulus was estimated as described above. The activation rates for LPSs concentrations 0.5, 5, and 50 pg/ml were calculated from a one-step decay function adjusted to pass through the experimental data that are also summarized in Table 5. Overall, these results showed that the LPS concentration highly impacted the activation rate (Table 5). Interestingly, no priming effect was observed in our system even with LPSs concentrations as low as 0.5 pg/ml (Figure 2H) or 0.05 pg/ml (data not shown). Graphic representation of the estimated activation rates from Table 5 in relation to the LPS concentration (Figure 3) showed a sigmoidal tendency that match with an activation Hill equation. Thus, the maximum activation rate, the AC50, and *n* parameters were obtained from the activation rate equation adjusted to fit the activation rates for each LPSs concentration (Figure 3) estimated from the experimental data shown in Table 5, which were set to 4.499/h, 0.1889 ng/ml, and 1.4825, respectively. Thus, the half-maximal activation rate takes place when the LPS concentration equals AC50, and the almost maximal rate occurs when LPS quadruples the value of this parameter.

Similarly to the activation rate, the TNF synthesis rate expressed as a Hill function depends on three parameters. To optimize the maximum TNF synthesis rate, the SC50 and the *m* parameters, the TNF synthesis rate was estimated using the Vensim’s optimization tool to fit the experimental data obtained from scenarios using various amounts of LPS (Figures 4A,B). As expected, the synthesis of TNF was highly dependent to the LPSs doses (Figure 4B) which led to different estimated TNF synthesis rates depending on the LPS concentration (Table 6). Representation of the estimated TNF synthesis rates (Table 6) showed a sigmoidal trend that indicated that the process can be explained by an activation Hill function (Figure 4C). The estimated values were used to adjust the Hill equation (Figure 4C) from which the maximum TNF synthesis rate, SC50, and *m* parameters were determined which were 0.0071 pg/h/monocytes, 0.1407 ng/ml, and 1.767, respectively. However, when these values were used to simulate the various scenarios, significant deviations between the model output and the experimental data were observed for lower LPSs doses (data not shown). In this sense, the values for maximum TNF synthesis rate and *m* were considered as definitive. However, the SC50 was further adjusted together with the immunomodulation rate, LPS removal rate, and TNF degradation rate parameters (Table 4) using a discrete model built in Matlab to obtain the best fitting considering all scenarios from Figure 4B. Using the adjusted values (Table 4), the model highly reproduces the shape of the TNF curves obtained after simulation of isolated monocytes from healthy volunteers with a wide range of LPSs doses (Figure 4B).

Similarly, to optimize the model parameters related to CCL2 synthesis and degradation, a Matlab discrete model was used to fit the data from an experimental ET model obtained, following the design described in Figure 5A. In agreement with what has been previously published (19), ET monocytes showed a reduced capacity to produce TNF whereas CCL2 was upregulated (Figures 5B,C). As with the initial value of LPSs in the medium, factors related to the initial characteristics of the monocyte subsets have been set as user-defined. Therefore, setting different values for these variables allows the simulation of particular scenarios, such as the response of ET monocytes to LPSs. To simulate the ET scenario, the model was programmed with 0, 75,000, and 925,000 of the initial monocyte population in the resting, proinflammatory, and ET states, respectively, and then simulated with initial concentration of 5 ng/ml of LPS. The TNF curves obtained after simulation of the control and ET scenarios were similar to those observed in the cultures of monocytes (Figure 5B) which prove the fitness of the model and validate the optimization process of the TNF synthesis parameters explained above. The parameters CCL2 synthesis rate on resting, CCL2 synthesis rate on inflammation, CCL2 synthesis rate on ET, and CCL2 degradation rate were automatically optimized to fit the CLL2 levels measured in monocytes cultures (Figure 5C). As expected, both the TNF and CCL2 curves obtained after simulation of the ET scenario were markedly different from those obtained in a scenario programmed with all the monocytes in the resting state (Figures 5B,C, compare black and blue lines). Thus, the model showed a lower production of TNF and higher production of CLL2 when simulating the ET scenario compared with the control conditions. These results indicate that this model is able to reproduce the ET phenomenon.

### Use of the Model: An Example with Septic Monocytes

Since initial monocyte population in the resting and ET levels can be considered dependent on each other, a tolerance index parameter was included in a modified version of the model. This parameter specifies the initial percentage of the initial monocytes that are in ET and is used to calculate the initial population in ET and resting states (Table 7). Since the transition of the monocytes through the proinflammatory state is a rapid process, the initial proinflammatory monocytes value can be considered equal to 0.

**Table 7:** Variables in the extended model.

*N*	Parameter	Equation/value range	Units
1	Tolerance index	0–100	Dimensionless
2	Total monocytes	User defined	Monocytes
3	Initial resting monocytes	$\text{Total monocytes}\cdot \frac{100–\text{Tolerance index}}{100}$	Monocytes
4	Initial ET monocytes	$\text{Total monocytes}\cdot \frac{\text{Tolerance index}}{100}$	Monocytes

To further analyze whether our model is able to simulate intermediate ET scenarios, monocytes from healthy volunteers were challenged with 0.5 ng/ml LPSs for 30 min and then incubated in fresh medium for 16 h allowing the culture to reach the ET (Figure 6A). Similar to results in Figure 5, this led to a decrease in the production of TNF and an increase in the production of CCL2 in comparison with the control scenario (Figures 6B,C, compare gray triangles with black circles). By setting the Tolerance index value in the model to 68%, the TNF and CCL2 outputs reproduce the cytokine curves obtained after a second LPS challenge with 5 ng/ml LPSs (Figures 6B,C), indicating that the model is able to reproduce different ET intensities in monocyte cultures.

We and other authors have previously reported that monocytes derived from patients with sepsis are in an ET state in which they show a diminished inflammatory response after *ex vivo* LPSs challenge (7, 8). Taking this into account, the model was programmed with a Tolerance index of 68%, and then simulated with initial concentration of 5 ng/ml of LPSs. The TNF and CCL2 levels at 16 h of simulation were similar to those observed in the cultures of monocytes isolated from patients with sepsis and challenged with 5 ng/ml LPSs for 16 h (Figures 6D,E) reinforcing the idea that this model is able to reproduce the ET exhibited by cultured monocytes from patients with sepsis when they are challenged with LPSs, and that it could be used to determine the tolerance status of the monocytes of these patients.

## Discussion

The immune system is able to initiate various types of responses, depending on the threat. Regulation of these responses involves the interaction of a high number of cell types, soluble mediators, and/or cell receptors. This complexity makes the immune system an excellent field in which to apply system dynamics, which has also been used for other biological science issues, such as exploring the dynamics of enzymatic reactions and improving the efficiency of bioreactors to be applied in the biotechnology industry (38, 39).

Monocyte/macrophage myeloid cells play major roles in the response of the innate immune system to endotoxins by initiating a protective inflammatory response that develops during time through various phases, from initiation and full inflammation to resolution, and re-establishment of tissue integrity. Although the response of monocytes to endotoxins and the ET phenomenon has been intensely studied in recent years, here we propose a mathematical model that improves our understanding, at a quantitative level, of this immunological process. The model considers that the endotoxin activates the resting monocytes that come into a transient inflammatory state, after which they move toward an ET condition. Because the inflammatory and ET monocytes produce various sets of cytokines in response to LPSs, we have included the levels of TNF as an inflammatory indicator and CCL2 as a tolerant marker to indirectly distinguish the monocyte phenotypes.

The accurate quantification of the parameters is critical to model validity. Therefore, we combined experimental information obtained from monocyte cultures challenged with LPSs with optimization processes to set the final values for the model parameters (Table 4). Among the factors influencing the response of the monocytes to endotoxins, the concentration of the endotoxin (e.g., LPSs) in the extracellular environment is known to be essential to the activation process (40). In the model, we assumed that the reduction in the extracellular LPSs led to a decrease in the rate of activation modulated by a Hill function, which has previously been used to reproduce activation activities in complex biological systems such as the binding of transcription factors to DNA (41). In addition, the TNF synthesis rate modulation by the LPS concentration is also described by an activation Hill equation. Consequently, the model can reproduce experimental challenges using a wide range of LPSs doses (Figure 4). In this sense, the model shows that the amplitude and shape of the inflammatory phase is directly proportional to the strength and the extent of the external stimuli, indicating that it can simulate a wide variety of biological situations. For example, whereas stimulation of naive monocytes with high-dose LPSs induces a strong but relatively short inflammatory phase (as measured by the production of TNFα), the challenge with low-dose LPSs provokes a significantly lower inflammatory phase (Figure 4). Interestingly, by programming a constant low-dose LPS (e.g., considering no LPS removal), the model is able to reproduce situations in which there is low-grade inflammation for long time periods (data not shown), as occurs in chronic diseases including obesity and diabetes, and in cardiovascular and neurodegenerative pathologies (42–45). Note that although there are differences in the goodness for each independent scenario in Figure 4B, this can be explained because of the adjustment was performed considering the four scenarios at the same time.

Pretreatment of murine macrophages with super-low doses of LPS has been shown to result in a more robust cytokine production to a secondary LPSs challenge (20–22). In contrast, *ex vivo* stimulation of human monocytes isolated from healthy volunteers with LPS doses as low as 0.5 pg/ml (Figure 2H) or 0.05 pg/ml (data not shown) did not significantly alter the response to a second LPSs challenge in the experimental system. In line with these results, Dillingh et al. proved that injection of very low doses of LPSs in human volunteers induced a reduction in TNFα and IL-1β production after an *ex vivo* LPS challenge to the isolated monocytes (24). This result can be explained by taking into account that humans are much more sensitive to LPSs than mice, and it strongly suggests that murine models are not the best option to study the response of human monocytes to endotoxins (23, 24).

This mathematical model has important implications for better understanding the dynamics of two physiological processes, such as the inflammatory response and ET in humans. Previous mathematical models were experimentally calibrated by using datasets obtained from experiments using animals (28, 29). Altogether, this and our previous models clearly suggest that although there are similarities, there are some differences in the response to endotoxins between humans and other animals. There are also implications in terms of simulating the response of monocytes to LPSs in a pathological scenario, such as that which occurs in sepsis, which is known to exhibit marked ET (7, 8) as a result of functional reprogramming mediated by hypoxia-inducible factor-1α (46). ET scenarios can be simulated by adapting the initial values of the three monocyte subsets (Figure 5) or incorporating in the model the Tolerance index parameter (Figure 6) which allows us to set the initial ratio between the monocytes in the resting and ET states. Interestingly, the simulation output can be easily adjusted to the measured cytokine levels by varying the value of this parameter. Therefore, the Tolerance index can be used to determine the ET status of the monocytes from septic patients. Given that ET is associated with increased mortality and nosocomial infections in patients with sepsis, the Tolerance index could be used as a way to predict the risk of developing these complications (47–49). Nevertheless, the incorporation of other indicators involved in the immune response into the model could improve the model as potential prognostic tool to be used in the clinical setting.

Interestingly, a good practice in system dynamics modeling is to start with a simple model, whose complexity can be progressively increased by including new processes. In this sense, our model is presented in a modular and hierarchical way, dividing the system into submodels with a similar level of complexity. This strategy facilitates the incorporation of new subsystems considering the production of other cytokines and/or other mediators that are able to impact on the response to endotoxins. Therefore, other proinflamatory indicators, such as IL-6 and IL-1β, or anti-inflammatory, such as IL-10 and TGF-β, can be easily included as new subsystems in our model. The parameters of these subsystems could be estimated following an experimental approach similar to the one used for TNFα and CCL2. Other example is that our model only considers LPS as stimulator but subsystems for other molecules affecting the activation flow of the monocytes might be incorporated. Experimentally this new process could be parameterized and optimized in a similar way that the one performed in Figures 2 and 3.

## Ethics Statement

This study was carried out in accordance with the recommendations of La Paz University Hospital Ethics Committee with written informed consent from all subjects. All subjects gave written informed consent in accordance with the Declaration of Helsinki. The protocol was approved by the La Paz University Hospital Ethics Committee.

## Author Contributions

EA, FM, and EL-C provided the idea and conceived and designed the experiments and supervised the work. EA, VT, FM, EH-J, CC-Z, AV-S, JC-M, JA-O, LA, FA, CM-E, AM-Q, and MD performed the experiments. EA, VT, FM, EH-J, CC-Z, and EL-C analyzed the data. EA and EL-C wrote the manuscript. All the authors revised and approved the manuscript.

## Conflict of Interest Statement

The authors declare that the research was conducted in the absence of any commercial or financial relationships that could be construed as a potential conflict of interest.
